# Generation of Schrödinger Cat States in a Hybrid Cavity Optomechanical System

**DOI:** 10.3390/e24111554

**Published:** 2022-10-29

**Authors:** Xingwei An, Tonghui Deng, Lei Chen, Saiyun Ye, Zhirong Zhong

**Affiliations:** Department of Physics, Fuzhou University, Fuzhou 350002, China

**Keywords:** hybrid cavity optomechanical system, quantum network

## Abstract

We present an alternative scheme to achieve Schrödinger cat states in a strong coupling hybrid cavity optomechanical system. Under the single-photon strong-coupling regime, the interaction between the atom–cavity–oscillator system can induce the mesoscopic mechanical oscillator to Schrödinger cat states. Comparing to previous schemes, the proposed proposal consider the second order approximation on the Lamb–Dicke parameter, which is more universal in the experiment. Numerical simulations confirm the validity of our derivation.

## 1. Introduction

Quantum physics has many distinguishing features from the classical counterpart. Among various characteristics, the superposition principle is the most striking one. It provides the possibility to associate with two states of the system through the arbitrary linear combination. One typical superposition states is the Schrödinger cat state [1,2], also referred to as the superposition of macroscopically distinguishable state of the single-mode quantized electromagnetic field. In particular, the even (+) and odd (−) coherent states defined by $|\Psi^{\pm }{\rangle =N\left(\alpha \right)}_{\pm }(|-\alpha \rangle \pm \left|\alpha \rangle \right)$, where $N{\left(\alpha \right)}_{\pm }$ is the normalization constant. These superposition states not only present quantum interference in phase space, but also play a significant role in the field of quantum information processing and are the central to explore the fuzzy quantum-classical boundary. A number of proposals have been proposed to achieve the Schrödinger cat states both in theories [3,4,5,6,7], and in experiments [8,9,10,11,12].

So far, most of the quantum phenomenon only exist in microscopic systems. However, according to the principles of quantum mechanics, there is nothing that forbids counterintuitive phenomena occur in mesoscopic distinct systems, such as entanglement or superposition. To reveal or observe these quantum phenomena, the key issue is to find a new platform which contains mesoscopic massive objects, such as mesoscopic mirrors. The cavity optomechanical system [13,14,15,16] comprising one fixed mirror and one movable mirror, is considered as a natural platform for experimental tests of fundamental quantum mechanics principles at large scales, and is considered as a promising approach for applications ranging from the detection of weak forces to small displacements, i.e., single molecule detection [17], gravitational wave interferometry [18,19,20,21], and precision sensing [22]. Also, this system is employed to study the fundamental transition between the quantum and classical world. Based on cavity optomechanical system, people have found some interesting quantum phenomenon, such as entanglement between mechanical oscillator and cavity mode [23,24,25,26,27], as well as between different mechanical oscillators or different cavity modes [28,29,30,31,32], and a quantum superposition state of a mechanical oscillator [33,34,35,36,37,38,39]. Note that the cavity optomechanical system has been put forward to study the quantum interference [40], electromagnetically induced transparency [41,42,43], dynamical casimir effect [44], and quantum Rabi model [45,46].

Generally, generating nonclassical states of macroscopic system is still a difficult task in nanomechanical systems, since the direct coupling efficiency between cavity mode and mechanical oscillator is relatively low which results in a long interaction time. It is pointed out that the introducing atoms or Kerr media into the cavity can effectively enhance coupling strength between the cavity and the mechanical oscillator [37,38,47,48]. An effective scenario to solve this drawback is that to introduce a quantum two-level system into the cavity optomechanical system. By using this method, Pirkkalainen et al., have demonstrated that the radiation–pressure interaction can be boosted to six order level. Cotrufo et al., have pointed out that the atom-phonon coupling rate can be improved by an optical driven field, which can be applied to the generation of nonclassical states of the mechanical oscillator [49]. Also, schemes have been presented to generate macroscopic Schrödinger cat states in the (hybrid) optomechanical system [50,51]. The scheme in Ref. [50], the macroscopic Schrödinger cat states of two mechanical resonators were generated by using a modulated photon-hopping interaction, which need a couple cavity system. Moreover, proposal has been presented to achieve macroscopic Schrödinger cat states swapping [51] and squeezed-coherent-cat state [52] in cavity optomechanical system.

**Figure 1:**
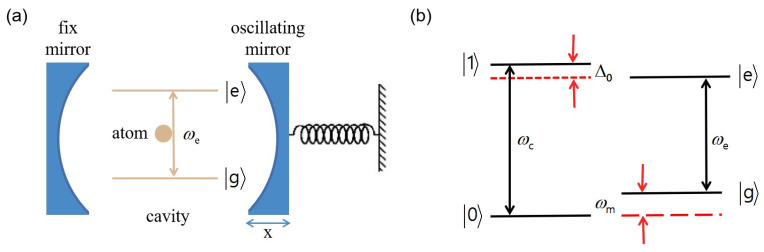
(**a**) Schematic diagram of a hybrid cavity-optomechanical system. A two-level atom is trapped in the cavity. The cavity mode simultaneously couples to the atom and the mechanical oscillators. (**b**) Energy levels of the atom for the resonant δ=0, where $\delta \equiv \omega_{c}-\omega_{e}-\Delta_{0}+\omega_{m}$.

In this paper, we propose a scheme for creating macroscopic Schrödinger cat states in a hybrid optomechanical system, as schematically in Figure 1a. Our proposal focuses on the single-photon strong-coupling regime, which require the coupling strength between cavity and mechanical oscillator is large enough. We show that, with suitable parameters, the photon-induced radiation pressure created by the atom-cavity interaction can drive the oscillator to macroscopic Schrödinger cat states. The significant difference between the present proposal and the previous ones [37,38,50] is that the second order approximation on the Lamb–Dicke parameter is considered, which is more universal to actual physical processes. Numerical simulations show that the fidelity of the cat states can reach.

The paper is organized as follows. In Section 2, we present the physical model, in which the mechanical oscillator is coupled to the cavity field driven by an atom. An effective Hamiltonian is derived to describe the interaction mechanism of this system in a single-photon strong-coupling regime. Such interaction mechanism directly induces the vibrational mode in Schrödinger cat states. In Section 3, numerical simulations of the fidelities of the generated states are discussed. Meanwhile, we compare the Wigner functions of the prepared states and corresponding ideal cat states. Finally, discussion and conclusion appear in Section 4.

## 2. Model and Hamiltonian

We consider the theoretical model based on a single-mode cavity with a movable perfectly-reflecting microsize mirror, which has mass *M*, position *q*, and vibrational frequency $\omega_{m}$. A two-level system (with the lowest levels denoted as |g〉 and the high one denoted as |g〉) is trapped in the cavity. The interaction between the atom and the cavity mode can be described by the standard Jaynes-Cummings model (JC) model, as sketched in Figure 1. In addition, the radiation pressure leads to the interaction between the cavity mode and the mechanical oscillators. The Hamiltonian of this system can be written as (ℏ=1)

(1) $$\begin{array}{c} H=H_{0}+H_{\mathrm{OM}}+H_{\mathrm{JC}}, \end{array}$$

where

(2) $$\begin{array}{c} H_{0}=\omega_{c}a^{†}a+\omega_{e}\left|e\rangle \langle e\right|+\omega_{m}b^{†}b, \end{array}$$

is the free Hamiltonian (assuming $\omega_{g}=0$)

(3) $$\begin{array}{c} H_{\mathrm{OM}}=g_{0}a^{†}a\left(b^{†}+b\right), \end{array}$$

is the normal optomechanical Hamiltonian, and

(4) $$\begin{array}{c} H_{\mathrm{JC}}=g(a|e\rangle \langle g|+a^{†}|g\rangle \langle e|), \end{array}$$

is the standard JC model. We consider the rotating wave approximation (RWA). Here *a*$\left(a^{†}\right)$ and *b*$\left(b^{†}\right)$ denote the annihilation (creation) operator for the cavity mode with frequency $\omega_{c}$ and the mechanical oscillator mode with frequency $\omega_{m}$, respectively. The parameter $\omega_{e}$ is the atomic transition frequency (|g〉 is denoted as the null-energy level) and the atom-cavity coupling strength is *g*. In addition, $g_{0}=\omega_{c}x_{\mathrm{zpf}}/L$ is the single-photon optomechanical coupling strength between the optical cavity and the mechanical oscillator, in which $x_{\mathrm{zpf}}=\sqrt{1/(2M\omega_{m})}$ describes the zero-point fluctuation of the moving mirror with mass *M*, and *L* is the length of the cavity.

We first perform a transformation $V_{1}=\exp\left[i\omega_{e}(a^{†}a+|e\rangle \langle e|)t\right]$ on the total Hamiltonian *H* of the system in Equation (1). The transformed Hamiltonian becomes

(5) $$\begin{array}{ccc} & H_{1} & =V_{1}HV_{1}^{†}-iV_{1}\frac{\partial V_{1}^{†}}{\partial t}\\ & & =\Delta a^{†}a+\omega_{m}b^{†}b+g_{0}a^{†}a\left(b^{†}+b\right)+g\left(a^{†}s^{-}+as^{+}\right), \end{array}$$

where $\Delta =\omega_{c}-\omega_{e}$ is the detuning between the cavity frequency $\omega_{c}$ and the atomic-transition frequency $\omega_{e}$. Then we introduce another unitary operator $V_{2}=\exp\left[\left(g_{0}/\omega_{m}\right)a^{†}a\left(b^{†}-b\right)\right]$ to transform the Hamiltonian $H_{1}$ into

(6) $$\begin{array}{cc} H_{2} & =V_{2}H_{1}V_{2}^{†}\\ & =\left(\Delta -\Delta_{0}a^{†}a\right)a^{†}a+\omega_{m}b^{†}b\\ & +g(a^{†}|g\rangle \langle e|e^{\eta (b^{†}-b)}+H.c.), \end{array}$$

where $\eta =g_{0}/\omega_{m}$ can be regarded as the Lamb–Dicke parameter, which is similar to the analysis in trapped ions [53,54]. The parameter $\Delta_{0}=g_{0}^{2}/\omega_{m}$ characterizes the nonlinearity of the cavity field induced by the mechanical oscillator. In the single-photon strong-coupling regime, the nonlinear parameter $\Delta_{0}=g_{0}^{2}/\omega_{m}$ is big enough so that photon blockade occurs, which effectively guarantees that the cavity modes are at two low energy levels, |0〉 and |1〉. Thus, the creation (annihilation) operator of the cavity field can be rewrite as $a^{†}=\left|1\rangle \langle 0\right|=\sigma_{+}$ ($a=\left|0\rangle \langle 1\right|=\sigma_{-}$), and the operator further introduce a new operator $\sigma_{z}=\left|1\rangle \langle 1\right|-\left|0\rangle \langle 0\right|$. Hence, one can rewrite the Hamiltonian $H_{2}$ as

(7) $$\begin{array}{c} H_{2}=H_{0}^{{}^{\prime }}+H_{i}, \end{array}$$

(8) $$\begin{array}{c} H_{0}^{{}^{\prime }}=\omega_{0}\frac{\sigma_{z}}{2}+\omega_{m}b^{†}b, \end{array}$$

(9) $$\begin{array}{c} H_{i}=g\left(\sigma_{+}|g\rangle \langle e|e^{\eta (b^{†}-b)}+H.c.\right), \end{array}$$

where $\omega_{0}=\Delta -\Delta_{0}$. The Hamiltonian in Equation (7) is analogous to the trapped ion system [55]; thus, $H_{i}$ becomes

(10) $$\begin{array}{c} H_{i}=g\sigma_{+}|g\rangle \langle e|\sum_{m=0,n=0}^{\infty }\frac{{\left(-1\right)}^{n}\eta^{(m+n})}{m!n!}{\left(b^{†}\right)}^{m}b^{n}+H.c. \end{array}$$

Considering the Lamb–Dicke approximation condition, we expand up to the second order expansion of η. The Hamiltonian in Equation (7) can be expressed as

(11) $$\begin{array}{c} H_{i}\approx g\sigma_{+}|g\rangle \langle e|\left[1+\eta \left(b^{†}-b\right)+\frac{\eta^{2}}{2}{\left(b^{†}-b\right)}^{2}\right]+H.c. \end{array}$$

In the interaction picture, we obtain

(12) $$\begin{array}{ccc} H_{i}^{\prime } & & =e^{iH_{0}^{{}^{\prime }}t}H_{i}e^{-iH_{0}^{{}^{\prime }}t}\\ & & =g\sigma_{+}|g\rangle \langle e|e^{i\omega_{0}t}[1+\eta \left(b^{†}e^{i\omega_{m}t}-be^{-i\omega_{m}t}\right)\\ & & -\eta^{2}b^{†}b+\frac{\eta^{2}}{2}{\left(b^{†}\right)}^{2}e^{2i\omega_{m}t}+\frac{\eta^{2}}{2}b^{2}e^{-2i\omega_{m}t}]+H.c. \end{array}$$

Under the condition $\omega_{0}=\Delta -\Delta_{0}$ = 0, $\omega_{m}\gg g,\eta g,\eta^{2}g$ we have

(13) $$\begin{array}{ccc} H_{i}^{\prime } & & =g(\sigma_{+}|g\rangle \langle e|+\sigma_{-}|e\rangle \langle g|)\\ & & -g\eta^{2}b^{†}b\left(\sigma_{+}|g\rangle \langle e|+\sigma_{-}|e\rangle \langle g|\right). \end{array}$$

Then, we can rewrite the operator $S^{+}=\sigma_{+}|g\rangle \langle e|$, $S^{-}=\sigma_{-}|e\rangle \langle g|$. Thus, we can obtain the approximate Hamiltonian

(14) $$\begin{array}{c} H_{eff}=H_{e1}+H_{e2}, \end{array}$$

(15) $$\begin{array}{c} H_{e1}=g\left(S^{+}+S^{-}\right), \end{array}$$

(16) $$\begin{array}{c} H_{e2}=-g\eta^{2}b^{†}b\left(S^{+}+S^{-}\right). \end{array}$$

such that $[H_{e1},H_{e2}]=0$. Clearly, all the essential dynamics is contained in

(17) $$\begin{array}{c} H_{e2}=-g_{e}b^{†}b\left(S^{+}+S^{-}\right), \end{array}$$

where $g_{e}=g\eta^{2}$, is effective coupling strength. In addition, the Equation (17) is the effective Hamiltonian of the system, which governs the evolution of the cavity field and the vibrational mode. Supposed the system is initially in the state $|\psi (0)\rangle =|g\rangle |0\rangle |\alpha \rangle$, which means that the atom, cavity field and mechanical oscillator are in the ground state $|g\rangle$, vacuum state $|0\rangle$, and coherent state $|\alpha \rangle$, respectively. In the rotating frame of $|u(t)\rangle =\exp\left[-iH_{e2}t\right]$, the evolution operator is written as

(18) $$U\left(t\right)=e^{-iH_{e2}t}=e^{ig_{e}b^{†}b\left(S^{+}+S^{-}\right)t},$$

the time evolution of system is

(19) $$\begin{array}{ccc} |\psi (t)\rangle & & =U\left(t\right)|\psi (0)\rangle \\ & & =e^{ig_{e}b^{†}b\left(S^{+}+S^{-}\right)t}|g\rangle |0\rangle |\alpha \rangle , \end{array}$$

After some mathematical manipulation [56] it is shown that

(20) $$\begin{array}{c} |\psi (t)\rangle =|g\rangle |0\rangle |\phi_{+}\rangle +|e\rangle |1\rangle |\phi_{-}\rangle , \end{array}$$

where $\phi_{\pm }=\frac{1}{2}\left(|\alpha e^{ig_{e}t}\rangle \right)\pm |\alpha e^{-ig_{e}t}\rangle )$ are superposition of two coherent states. The state of Equation (20) is an entangled state of the cavity and mechanical oscillator at $g_{e}t=k\pi /2$ (*k* is arbitrary integer number). If $g_{e}t=\pi /2$, Equation (20) becomes

(21) $$\begin{array}{c} |\psi \rangle =\frac{1}{2}\left[|g\rangle |0\rangle \left(|i\alpha \rangle +|-i\alpha \rangle \right)\right]+|e\rangle |1\rangle \left(|i\alpha \rangle -|-i\alpha \rangle \right)], \end{array}$$

Thus, we can obtain the even and odd coherent states (the Schrödinger cat states) by measuring the state of the atom. If the atom is detected in the ground state (excited state), the state of the mechanical mode is $|\phi_{cat}^{+}\rangle =N_{+}\left(|i\alpha \rangle +|-i\alpha \rangle \right)$ ($|\phi_{cat}^{-}\rangle =N_{-}\left(|i\alpha \rangle -|-i\alpha \rangle \right)$). Here, the state $|\phi_{cat}^{+}\rangle$ and $|\phi_{cat}^{-}\rangle$ is the so called even and odd coherent states (the Schrödinger cat states)

(22) $$\begin{array}{c} |\phi_{cat}^{\pm }\rangle =N_{\pm }\left(|i\alpha \rangle \pm |-i\alpha \rangle \right), \end{array}$$

with normalization constants

(23) $$\begin{array}{c} N_{\pm }={\left[2\left(1\pm e^{-2{\left|\alpha \right|}^{2}}\right)\right]}^{-1/2}, \end{array}$$

The probability is given in the following

(24) $$\begin{array}{c} P_{\pm }=\frac{1}{2}\left(1\pm e^{-2{\left|\alpha \right|}^{2}}\right). \end{array}$$

## 3. Numerical Simulations

The validity of above derivations can be verify by numerical simulation. In this section, we firstly compare the dynamics governed by effective Hamiltonian in Equation (17) and by the full Hamiltonian in Equation (1). Defining the Lindblad–Kossakowski superoperator $D\left[o\right]\rho =\left(1/2\right)\left(2o\rho o^{†}-o^{†}o\rho -\rho o^{†}o\right)$, the master equation for the system is given by

(25) $$\begin{array}{ccc} & \dot{\rho }= & -i\left[H^{{}^{\prime }},\rho \right]+\kappa D\left[a\right]\rho +\gamma D\left[s\right]\rho \\ & & +\gamma_{1}\left\{\left(n_{\mathrm{th}}+1\right)D\left[b\right]\rho +n_{\mathrm{th}}D\left[b^{†}\right]\rho \right\}, \end{array}$$

where ρ is the density operator of the system, s=|g〉〈e| is the atomic transition operator, $H^{{}^{\prime }}=H$ and $H^{{}^{\prime }}=H_{e2}$ correspond to the full Hamiltonian in Equation (1) and effective Hamiltonian in Equation (17), κ, γ and $\gamma_{1}$ are the decaying rates of the cavity, atom and oscillator, respectively. For simplicity, we assume that the mechanical oscillator couples to a thermal reservoir with temperature *T*, and the corresponding thermal phonon number is $n_{\mathrm{th}}={\left[\exp(\hbar \omega_{m}/k_{B}T)-1\right]}^{-1}.$ Although the atom-cavity coupling strength depends on the atomic position in the cavity, under the current technology, the atom has trapped at the single-photon level so that the effect of atomic center-of-mass on the motion on the coupling strength can be neglected [57,58,59]. Therefore, in all subsequent numerical simulations, the atom-cavity coupling coefficient is considered as a constant. In the simulation, the parameters are chosen as follows: g=3.199, $g_{0}=0.8$, $\omega_{c}=200\pi$, $\omega_{m}=25$, α=4, κ=0.01, $\gamma =\gamma_{1}=0.001$ and $n_{\mathrm{th}}=1$.

**Figure 2:**
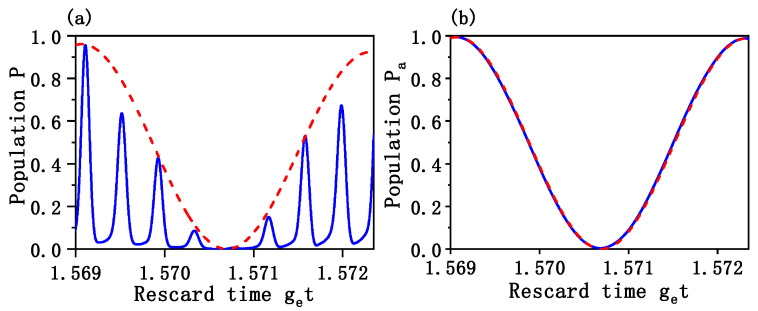
(**a**) The time evolution of the population $P_{0}=|\langle \psi \left(0\right)|\psi (t)\rangle {|}^{2}$ of the proposed initial state $|\psi (0)\rangle =|g\rangle |0\rangle |\alpha \rangle$ governed, respectively, by the effective Hamiltonian (red-dash curve) and the full Hamiltonian (blue solid curve). (**b**) The time evolution of the population $P_{a}=\langle \psi \left(t\right)|\sigma^{†}\sigma |\psi \left(t\right)\rangle$ of atom, where $\sigma^{†}=|g\rangle \langle e|$, $\sigma =|e\rangle \langle g|$. The corresponding parameters are set to be: g=3.199, $g_{0}=0.8$, $\omega_{c}$ = 200π, $\omega_{m}$ = 25, α = 4, κ=0.01, $\gamma =\gamma_{1}=0.001$ and $n_{\mathrm{th}}=1$.

Figure 2a shows the time evolution of the population $P={\left|\langle \psi (0)|\psi (t)\rangle \right|}^{2}$ of the proposed initial state $|\psi (0)\rangle =|g\rangle |0\rangle |\alpha \rangle$, in which the red-dash curve describes the population governed by the effective Hamiltonian and the blue solid curve describes the population governed by the full Hamiltonian. One can see from Figure 2a that the two curves are nearly consistent with each other at highest points, which indicates that the dynamic processes driven by these two Hamiltonians coincide roughly. Figure 2b shows the time evolution of the population $P_{a}=\langle \psi \left(t\right)|\sigma^{†}\sigma |\psi \left(t\right)\rangle$ of the initial state of the atom, where $\sigma^{†}=|g\rangle \langle e|$, and $\sigma =|e\rangle \langle g|$. It is obvious that the approximations adopted when deriving the effective Hamiltonian are valid, since the two curves described by the full and effective Hamiltonians nearly coincided.

The fidelity of the prepared $|\phi_{cat}^{\pm }\rangle$ state is defined as $F_{\pm }={\left|\langle \phi_{cat}^{\pm }|{\mathrm{Tr}}_{a,c}\left[\rho \left(t\right)\right]|\phi_{cat}^{\pm }\rangle \right|}^{2}$, where $Tr_{a,c}$ denotes the trace of the atom and cavity field. In order to obtain the target states, the projective measurement on the atom or cavity field is require. If the atom (the cavity) is detected the ground state (vacuum state), the system collapses to state $|\phi_{cat}^{+}\rangle$. Otherwise, if the atom (the cavity) is detected the excited state ( Fock state $|1\rangle$ ), the system collapses to state $|\phi_{cat}^{-}\rangle$.

**Figure 3:**
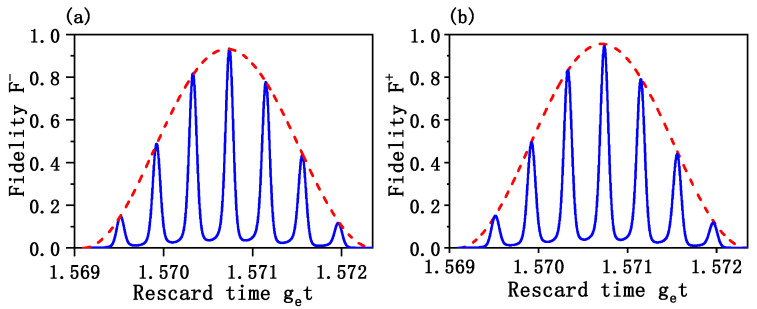
(**a**) Time evolution of the fidelity $F_{-}={\left|\langle \phi_{cat}^{-}|{\mathrm{Tr}}_{a,c}\left[\rho \left(t\right)\right]|\phi_{cat}^{-}\rangle \right|}^{2}$, governed, respectively, by the effective Hamiltonian (red-dash curve) and the full Hamiltonian (blue solid curve). The target odd cat state with fidelity higher than 0.950 (the effective Hamiltonian) and 0.952 (the full Hamiltonian) can be obtained at the time $t≃1.5708/g_{e}$. (**b**) Time evolution of the fidelity $F_{+}={\left|\langle \phi_{cat}^{+}|{\mathrm{Tr}}_{a,c}\left[\rho \left(t\right)\right]|\phi_{cat}^{+}\rangle \right|}^{2}$, governed, respectively, by the effective Hamiltonian (red-dash curve) and the full Hamiltonian (blue solid curve). The target even cat state with fidelity higher than 0.972 (the effective Hamiltonian) and 0.963 (the full Hamiltonian) can be obtained at the time $t≃1.5708/g_{e}$. The parameters are the same as Figure 2.

In Figure 3, we plot the time evolution of the fidelity of target states governed, respectively, by the effective Hamiltonian (red-dash curve) and the full Hamiltonian (blue solid curve). The parameters are the same with Figure 2. The numerical result shows that the fidelity of the odd cat state is higher than 0.950 for effective Hamiltonian and 0.952 for the full Hamiltonian at the time $t≃1.5708/g_{e}$, as is shown in Figure 3a. The theoretical value shows that the maximal fidelity appear at $g_{e}t=\pi /2≃1.5708$, which is in good agreement with the result of numerical simulation. In Figure 3b, one can see that the fidelity of the even cat state higher than 0.972 for the effective Hamiltonian and 0.963 for the full Hamiltonian at the time $t≃1.5708/g_{e}$, respectively. Thus, it is possible to drive the oscillator to the macroscopic Schrödinger cat states with a high fidelity.

**Figure 4:**
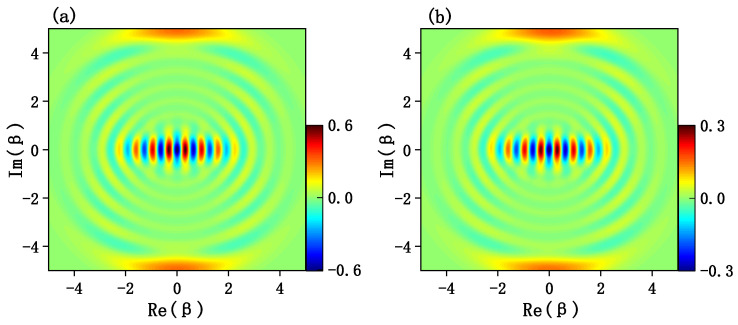
(**a**) Plane cut of the Wigner function W(β) for the ideal odd cat state $N^{-}\left(|i\alpha \rangle -|-i\alpha \rangle \right)$ with α=4. (**b**) Plane cut of the Wigner function W(β) obtained by the generated state at time $t=\pi /2g_{e}(≃$1.5708/$g_{e})$. The parameters are the same with Figure 2.

**Figure 5:**
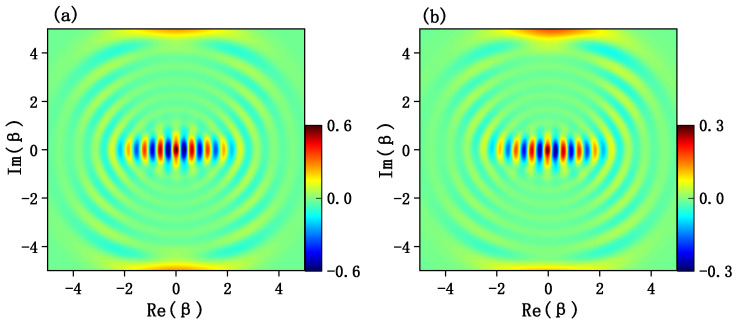
(**a**) Plane cut of the Wigner function W(β) for the ideal even cat state $N_{+}\left(|i\alpha \rangle +|-i\alpha \rangle \right)$ with α=4. (**b**) Plane cut of the Wigner function W(β) obtained by the generated state at time $t=\pi /2g_{e}(≃$1.5708/$g_{e})$. The parameters are the same as Figure 2.

Since the Wigner functions are usually used to character the quantum interference effect and quantum coherence effects of cat states in the phase space, it is necessary to calculate the joint Wigner functions for the target cat states. For a quantum state of a mechanical resonator described by a density matrix $\rho_{r}$, the Wigner function is defined by

(26) $$\begin{array}{c} W\left(\beta \right)=\frac{2}{\pi }Tr\left[D^{†}\left(\beta \right){\left(-1\right)}^{b^{†}b}\rho_{r}D\left(\beta \right)\right]. \end{array}$$

where $D\left(\beta \right)=\exp\left(\beta b^{†}-\beta^{*}b\right)$ is the displacement operator and $\rho_{r}$ is the density operator of the mechanical resonator. In Figure 4a, we calculate the plane cut of the Wigner function along the Re(β)-Im(β) axes for the ideal odd cat state $N_{-}\left(|i\alpha \rangle -|-i\alpha \rangle \right)$ with α=4, the corresponding Wigner function for target state at time $t=\pi /2g_{e}(≃$1.5708/$g_{e})$ is shown in Figure 4b. As we can see that both figures show fringes with alternating positive and negative values on the Re(β)-Im(β) plane cut, which indicate the quantum interference of the quantum state. In addition, the same Wigner functions for the ideal even cat state and the generated state at time $t=\pi /2g_{e}(≃$1.5708/$g_{e})$ are shown in Figure 5a and Figure 5b, respectively. As expected, the Wigner functions along the Re(β)-Im(β) axes obtained from the generated states are in agreement with result for the corresponding Schrödinger cat states.

## 4. Discussion and Conclusions

It is necessary to give a brief discussion of the experimental feasibility of the proposed scheme. Firstly, the two-level approximation of the cavity in the scheme can be guaranteed by the photon blockade effect, which has been demonstrate in the cavity optomechanical systems [60]. In addition, the proposed scheme requires the system work in single-photon strong-coupling regime, which require single-photon strong-coupling strength $g_{0}$ is much higher than the decay rates of cavity κ and mechanical oscillator $\gamma_{1}$. Secondly, the parameters we used in the simulation can refer to relevant experiments [47,61,62]. As reported in Ref. [47], the frequency of cavity is $\omega_{c}=2\pi \times 4.93$ GHz, the decay rate of the cavity is κ=2π×215 KHz, the frequency of mechanical oscillator is $\omega_{m}=2\pi \times 65$ MHz, and the corresponding mechanical damping rate is $\gamma_{1}=2\pi \times 15$ kHz, while the single-photon optomechanical coupling strength is $g_{0}=2\pi \times 1.6$ MHz. Thus, we think the proposed proposal may be realized in the near future as experimental techniques improved quickly.

In summary, we have proposed a scheme to generate Schrödinger cat states of a vibrating micro-size mirror (treat as a mechanical oscillator) with the assistance of a two-level atom and radiation pressure in a hybrid cavity optomechanical systems. Under the Lamb–Dicke limit and the suitable setting of the parameters, the radiation pressure induced by the photon created by the atom-cavity interaction can drive the oscillator to the macroscopic Schrödinger cat states. After numerically simulations, the results show the validity of our proposed scheme. And the measurement of the Schrödinger cat states can be realized by measuring the number of photons in the cavity in a quantum nondemolition measurement [63] or by detecting the state of the atom. Thus, the proposed scheme could be realized in the future. 

## Data Availability

Not applicable.
